# The role of TSC2 in breast cancer: a literature review

**DOI:** 10.3389/fonc.2023.1188371

**Published:** 2023-05-12

**Authors:** Qiao-Yan Zhu, Zhe-Min He, Wen-Ming Cao, Bei Li

**Affiliations:** ^1^ The Second Clinical Medical College of Zhejiang Chinese Medical University, Hangzhou, China; ^2^ Department of Breast Medical Oncology, Cancer Hospital of the University of Chinese Academy of Sciences (Zhejiang Cancer Hospital), Institute of Cancer and Basic Medicine (ICBM), Chinese Academy of Sciences, Hangzhou, China; ^3^ Department of Geriatric, Affiliated Hangzhou First People’s Hospital, Zhejiang University School of Medicine, Hangzhou, China

**Keywords:** TSC2, breast cancer, signaling pathways, mTOR, PI3K

## Abstract

*TSC2* is a tumor suppressor gene as well as a disease-causing gene for autosomal dominant disorder tuberous sclerosis complex (TSC). Research has found that some tumor tissues have lower TSC2 expression levels than normal tissues. Furthermore, low expression of TSC2 is associated with poor prognosis in breast cancer. TSC2 acts as a convergence point of a complex network of signaling pathways and receives signals from the PI3K, AMPK, MAPK, and WNT pathways. It also regulates cellular metabolism and autophagy through inhibition of a mechanistic target of rapamycin complex, which are processes relevant to the progression, treatment, and prognosis of breast cancer. In-depth study of TSC2 functions provides significant guidance for clinical applications in breast cancer, including improving the treatment efficacy, overcoming drug resistance, and predicting prognosis. In this review, protein structure and biological functions of TSC2 were described and recent advances in TSC2 research in different molecular subtypes of breast cancer were summarized.

## 1 Introduction

Loss-of-function mutations in *TSC1* or *TSC2* can give rise to an autosomal dominant disorder of tuberous sclerosis complex (TSC) that invades multiple organs, such as brain, skin, heart, lungs, and kidneys (1, 2). Moreover, *TSC1*/*2* are significant anti-oncogenes that are mutated in breast (3), liver (4–6), lung (7, 8), kidney (9, 10) and other (11) cancers. Allison et al. (3) have reported on the prevalence of *TSC1* germline mutations in 5,041 patients with breast cancer (pathogenic variants (PVs) vs. variants of uncertain significance (VUS): 0.00% vs. 0.30%) and the prevalence of *TSC2* germline mutations in 5,040 patients with breast cancer (PVs vs. VUS: 0.00% vs. 1.20%). Mehta et al. (12) have shown that *TSC1* rs7874234 was associated with delayed age at diagnosis of estrogen receptor (ER)-positive breast cancer (P = 0.0049). Daniel et al. have reported on 13 *TSC2* mutations and six *TSC1* mutations in 111 hepatocellular carcinomas cases (6). Moreover, 22% of 86 human lung cancer specimens were identified as those with *TSC1* and/or *TSC2* loss of heterozygosity (7). Furthermore, Yong et al. have shown that *TSC2* rs30259G>A was associated with worse overall survival and disease-free survival (DFS) in patients with non-small cell lung cancer after curative surgery (P < 0.05) (8). These findings suggested that *TSC1*/*2* may be candidates for a prognostic marker for several tumors.

Evidence of low TSC1/2 expression in prostate cancer (13) is lacking. However, studies have reported that the level of TSC1/2 expression is lower in breast cancer (12, 14) and oral squamous cell carcinoma (15) than in normal tissues. Data from a 10-year follow-up study have indicated that tumor tissues from breast cancer patients who relapsed or died had significantly low levels of TSC2 compared to patients who did not experience recurrence (P = 0.03 and 0.05, respectively). This suggested that low TSC2 expression was associated with aggressiveness and unfavorable prognosis in patients with breast cancer (14). TSC2-null breast cancer cells are sustained mechanistic targets of rapamycin complex 1 (mTORC1) activity that regulates essential cellular activities. Hyperactivated mTORC1 promotes the growth and metastasis of breast cancer (16–18). There is also growing evidence that TSC2 plays an essential role in breast cancer. Hence, the present review summarizes the functions of TSC2 and recent advances in TSC2 research in different subtypes of breast cancer in order to provide new directions for breast cancer clinical applications.

## 2 TSC2 structure

*TSC2* in chromosome 16p13.3 encodes a 200-kDa protein tuberin containing 1,807 amino acids (19). The GTPase-activating protein (GAP) catalytic domain located on the TSC2 C-terminal is a highly conserved domain that can combine with two Ras homologs enriched in the brain (Rheb) to convert Rheb from GTP-bound to GDP-bound (20, 21). This domain is homologous to Ras-proximate1 (Rap1) GAPs, suggesting that the mechanisms of Rheb-GTP hydrolysis are identical to those of the Rap-Rap1GAP system, which may rely on the asparagine thumb (N1643) to stabilize the γ-phosphate of GTP and promote GTP hydrolysis of Rheb (22, 23). The presence of a structural domain bound to TSC1 at the N-terminal of TSC2 and TSC1 plays a role in stabilizing TSC2 and preventing its degradation (22, 24, 25). TSC1, TSC2, and Tre2-Bub2-Cdc16 (TBC) 1 domain member 7 (TBC1D7) assemble to form the TSC multiprotein complex (TSCC) with a 2:2:1 stoichiometry (23, 26). One TBC1D7 concurrently interacts with two parallel TSC1 helices, indicating that TBC1D7 may react by binding with the C-terminal ends of TSC1 to stabilize the TSCC (27). TBC1D7 knockdown affects the link between TSC1 and TSC2, causing a decrease in GAP activity, but has no effect on the localization of TSC2 in the lysosome (26). Thus, the function of TSC2 is closely related to the structural integrity of TSCC.

## 3 TSC2 functions

### 3.1 TSC2 with mTORC

The mTORC1 acts as a protein kinase complex and is almost ubiquitous in eukaryotic cells. It regulates the nutritional status, cellular metabolism, and cell growth, including the synthesis of proteins, lipids, and nucleic acids (28, 29). The mTORC1 promotes mRNA transcription *via* phosphorylation of ribosomal protein S6 kinase and eukaryotic initiation factor 4E-binding protein 1 (30). It also prevents autophagy by regulating the lysosomal degradative capacity of cells or direct inhibition of the early autophagy process stages (31). TSC2 acts as an inhibitor of mTORC1 because its activation is dependent on Rheb-GTP (32). When TSC2 is absent or inactivated, the process of Rheb-GTP conversion to Rheb-GDP is blocked, leading to an increase in the level of Rheb-GTP to activate mTORC1 (33). Lysosomes are generally recognized as the main signaling platform for TSCC to inhibit mTORC1. In the absence of stress granules (SGs), the ras-GTPase-activating protein SH3-domain-binding protein (G3BP1/2) resides on the surface of the lysosomes and acts as a tether connecting TSCC (34). The N-terminal of the NTF2L domain of G3BP1 bonds with the lysosome-associated membrane proteins 1/2, which bridge the TSCC to the lysosomal surface (35). Thus, when conditions are beneficial for cell growth, TSC2 is diffusely localized in the cytoplasm. The amount of TSCC in the lysosome then decreases, while the abundance of Rheb-GTP increases, giving rise to the activated mTORC1 (33, 36). A single inhibitory stress factor, such as hypoxia and low amino acid levels, can cause lysosomal TSC2 repositioning to inhibit mTORC1 activation toward suppression of cell growth and promotion of autophagy (37). Compared to mTORC1, there are fewer studies related to the mechanism and function of TSC2 regulation of mTORC2. Some studies have found that TSC2 deletion can induce mTORC2-specific upregulation of Ras homolog family member A (38). Some studies have also found that TSC2 deletion impaired the enzymatic activity of mTORC2 (26). However, how TSC2 is involved in the regulation of mTORC2 is unclear and requires in-depth study.

### 3.2 TSC2 is the hub of complex signaling pathways

**Figure 1:**
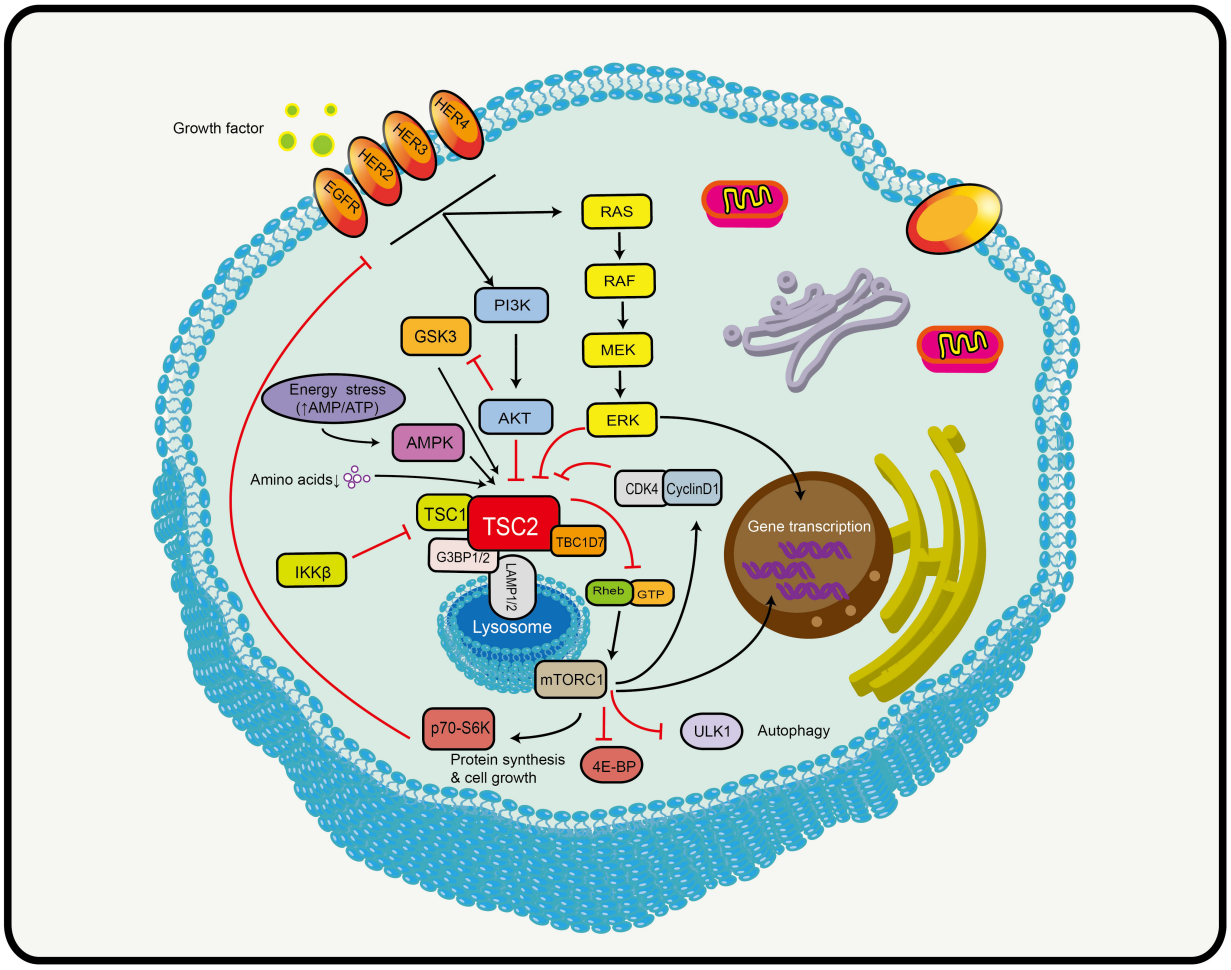
Schematic model depicting the TSC2-mTOR axis. TSC2 is the convergence point of a complex network of signaling pathways that receives signals from multiple signaling pathways, including PI3K, AMPK (AMP activated protein kinase), MAPK (mitogen-activated protein kinase, and WNT (see text for details). Black arrows indicate stimulation and red blocked arrows indicate inhibition. Abbreviations: HER2/3/4, human epidermal growth factor receptor 2/3/4; PI3K, phosphatidylinositol 3-kinase; AKT, protein kinase B; TSC1/2, tuberous sclerosis protein complex 1 and 2; TBC1D7, Tre2-Bub2-Cdc16 (TBC) 1 domain member 7; G3BP1/2, ras-GTPase-activating protein SH3-domain-binding protein; LAMP1/2, lysosomal-associated membrane proteins 1/2; GTP, guanosine triphosphate; Rheb, Ras homolog enriched in brain; mTORC1, mechanistic target of rapamycin complex 1; ULK1, unc-51-like kinase 1; 4E-BP1, 4E binding protein 1; p70s6k, ribosomal protein kinase S6; AMPK, AMP-activated protein kinase; GSK3, glycogen synthase kinase 3; RAS, rat sarcoma; MEK, mitogen-activated protein kinase; ERK, extracellular signal-regulated kinase; CDK4, cyclin D-dependent kinase 4.

TSC2 is the convergence point of a complex network of signaling pathways, where almost all upstream stimuli (including amino acids) that regulate mTORC1 activity converge on TSC2 (**Figure 1**) (37). The most well-known is the phosphoinositide-3-kinase (PI3K)/protein kinase B (AKT)/TSC2/mTOR pathway that insulin and other growth factors are dependent on (39, 40). The serine/threonine kinase AKT is located upstream of TSC2 and can directly phosphorylate the Ser939 and Thr1462 of TSC2. TSC2 is bound by the 14-3-3 protein in response to AKT phosphorylation and is then sequestered away from TSC1 (39). The 14-3-3 protein mediates the subcellular TSC2 translocation and protein hydrolytic degradation to increase the abundance of Rheb-GTP in the lysosome and thus promote mTORC1 activation (41). Hypoxia induces the regulated in development and DNA damage response (REDD1) to bind the 14-3-3 protein, releasing TSC2 from 14-3-3 (42). In addition, TSC2 is also involved in some classical signaling pathways, such as RAS and WNT signaling. The extracellular signal-regulated kinase 1/2 (ERK1/2) downstream of RAS signaling can phosphorylate the S664 of TSC2, leading to TSC1-TSC2 dissociation (43). In addition, S664 is a marker for ERK-mediated (rather than AKT-mediated) mTOR activation, which can be used to screen patients who may benefit from mTOR inhibitor (44). The p90 ribosomal S6 kinase acts as a downstream effector of ERK1/2 that inactivates TSC2 by phosphorylating it at a regulatory site Ser1798 (45). In hypoxic conditions and when energy deficiency was present, decreasing levels of intracellular ATP led to a rapid activation of AMP-activated protein kinase (AMPK) and TSC2 phosphorylation and enhancement of its activity (46, 47). This is the opposite of the effects of PI3K-AKT signaling in causing phosphorylation and inactivation of TSC2. Glycogen synthase kinase 3 (GSK3) downstream of WNT acts synergistically with AMPK. It can also phosphorylate TSC2 to increase its activity to inhibit mTOR (48). Based on this evidence, TSC2 plays an important role in cellular activities.

### 3.3 Other TSC2 functions

There are many forms of post-translational protein modification. Based on the above evidence, it is clear that TSC2 activity is regulated through phosphorylation, but is not completely dependent on it. TSC2 can also be acetylated by arrest defective 1 (ARD1), also known as N-α-acetyltransferase 10. ARD1 has a role in inhibiting cell proliferation, inducing autophagy, and suppressing tumor growth, which is associated with ARD1 acetylation, stabilization, and degradation inhibition of TSC2 (49). Seishu et al. (50) have found that protein arginine methyltransferase 1 methylates TSC2 at R1457 and R1459. The methylation of two residues inhibits phosphorylation of TSC2 at T1462, which is critical for TSC2 stability. Therefore, studies on the regulation of TSC2 activity might play a role in the development of treatment strategies.

TSC2 is a multifunctional protein that also regulates pathways beyond the mTOR pathway (51, 52). Loss-of-function TSC2 is able to activate Rac1 and thereby increase ROS production (53). TSC2 also plays a part in cell cycle regulation. It has been shown that TSC2 positively correlates with the expression of cyclin-dependent kinase inhibitor p27 in the epithelium (54). Down-regulation of TSC2 expression induced quiescent fibroblasts in G0 phase to re-enter the cell cycle (55). This may be related to the fact that TSC2 affects the stability and intracellular spatial distribution of p27 (56, 57). In addition, TSC2 can play the role of a transcription factor, inhibiting the expression of epidermal growth factor family member epidermal regulator epiregulin by binding to its promoter (58). TSC2 is extremely significant in modulating cell growth and proliferation, while its nuclear function remains to be further explored.

## 4 TSC2 in breast cancer

**Figure 2:**
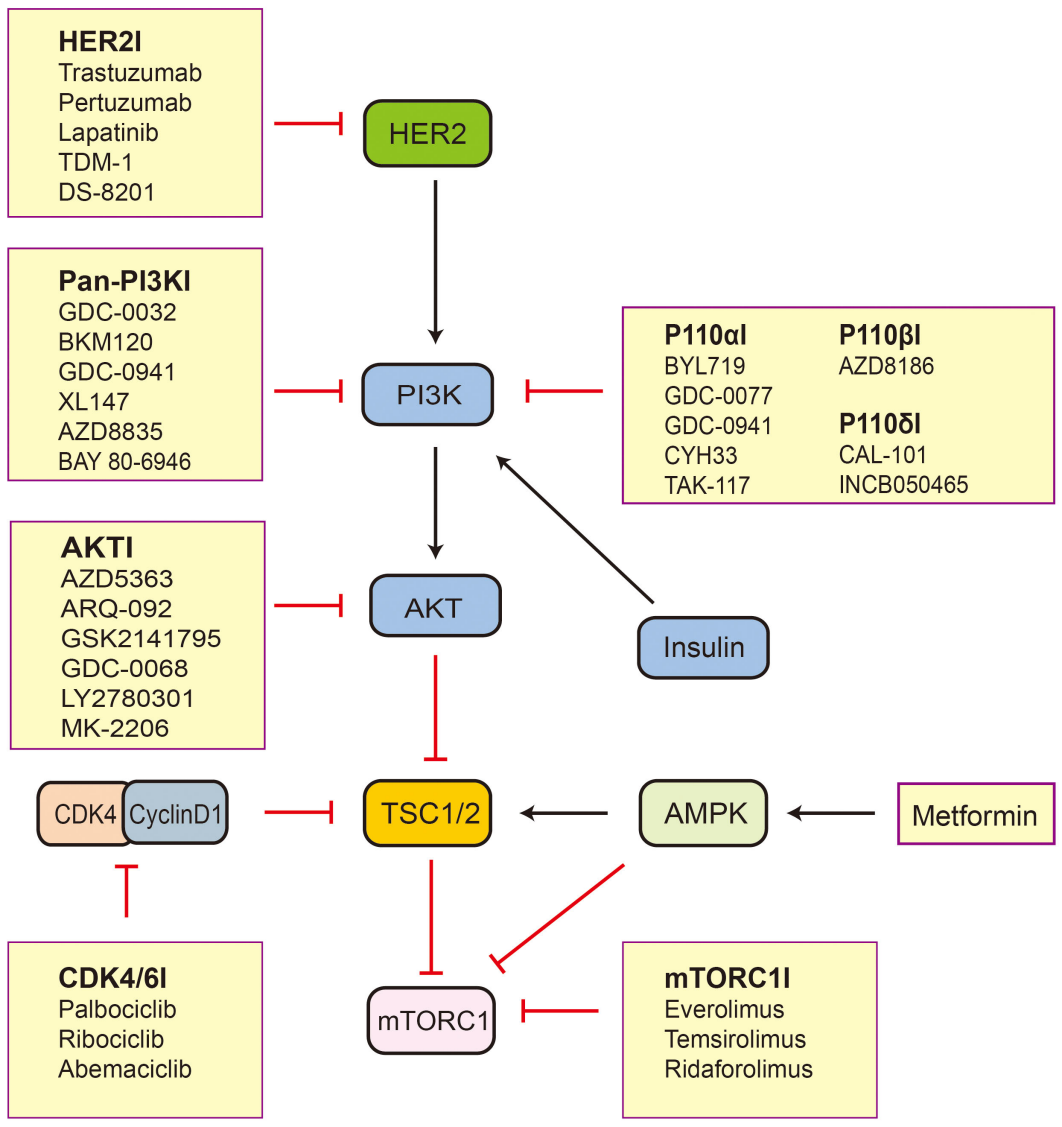
The inhibitors related to TSC2-mTOR axis active in preclinical and clinical trials. Black arrows indicate stimulation and red blocked arrows indicate inhibition. Abbreviations: HER2, human epidermal growth factor receptor 2; PI3K, phosphatidylinositol 3-kinase; AKT, protein kinase B; TSC1/2, tuberous sclerosis protein complex 1 and 2; mTORC1, mechanistic target of rapamycin complex 1; AMPK, AMP-activated protein kinase; CDK4, cyclin D-dependent kinase 4.

Breast cancer is the most common malignant tumor in females worldwide, with its incidence surpassing that of lung cancer in 2020 (59). Based on the expression of ER, progesterone receptor (PR), and human epidermal growth factor receptor 2 (HER2), breast cancer can be classified into three major subtypes that include hormone receptor (HR)-positive breast cancer, HER2-positive breast cancer, and triple-negative breast cancer (TNBC). There are significant differences in biological behavior, clinical manifestations, and treatment strategies among these subtypes, providing important information for clinical antitumor therapy (60). The TSC/mTOR pathway is frequently deregulated in breast cancers and is associated with tumorigenesis (61). The hypermethylation of TSC1 promoters can be observed in breast cancer (14). Lee et al. (62) have found that IKKbeta phosphorylates TSC1 at Ser487 and Ser511 to activate mTOR. The expression of activated IKKbeta correlates with TSC1 Ser511 phosphorylation and VEGF production in breast cancer patients and is associated with poor prognosis in breast cancer. It follows that TSC1 plays an important role in breast cancer. TSC2 acts as an integrator of various signaling cues and regulates cellular metabolism and autophagy through inhibition of mTORC1, which are processes relevant to the progression, treatment (**Figure 2**), and prognosis of breast cancer (37). Therefore, it is important to explore the function of TSC2 in breast cancer.

### 4.1 TSC2 in HER2-positive breast cancer

The ERBB family includes epidermal growth factor receptor (EGFR) (ERBB1), HER2 (ERBB2), HER3 (ERBB3), and HER4 (ERBB4), which are stimulated by ligands to form homodimers or heterodimers to activate intracellular pathways that are part of the RAS/RAF/MEK/ERK and PI3K/AKT/TSC2/mTOR pathways, promoting proliferation and metastasis of tumor cells (63). HER2 is overexpressed in approximately 15–20% of invasive breast cancers (64). HER2 does not directly bind with any ERBB ligands, but its conformation favors dimerization. Therefore, when HER2 is overexpressed, it can activate the downstream pathways through its own dimerization or heterodimerization of the other members of the ERBB family that bind to the ligands (65–67). EGFR can signal to PI3K through GAB1 in a HER3-independent manner, phosphorylating TSC2 and activating mTOR (68). There is a variety of clinical anti-HER2-targeting drugs. Multiple mechanisms of anti-HER2 therapy resistance have been demonstrated, with overactivation of the downstream PI3K/AKT/TSC2/mTOR pathway being the most common (69). Therefore, it is extremely significant to inhibit this pathway in anti-HER2 therapy. Inhibitors of cyclin D-dependent kinase 4/6 (CDK4/6) have been shown to reverse resistance to anti-HER2 therapy. CDK4 promotes cell proliferation both directly through retinoblastoma phosphorylation and indirectly through mTORC1 activation by binding and phosphorylating TSC2 on Ser1217 and Ser1452 to promote cell growth (70). CDK4/6 inhibition partially attenuates mTORC1 activity by reducing TSC2 phosphorylation, thereby relieving feedback inhibition of upstream ERBB family kinases and making tumors re-sensitized to EGFR/HER2 blocking. Therefore, dual inhibition of EGFR/HER2 and CDK4/6 more effectively inhibits TSC2 phosphorylation and blocks mTORC1 activity (71). Clinical research on CDK4/6 inhibitors combined with trastuzumab is underway (72). Metformin is an antidiabetic agent that also holds great promise in breast cancer treatment (73). It can not only indirectly lower the internal insulin levels but can also activate the AMPK pathway in the liver by depleting energy. Insulin relies on AKT-mediated phosphorylation of TSC2 to activate mTORC1, while AMPK enhances TSC2 activity and phosphorylates raptor on Ser722 and Ser792 in inhibiting mTORC1 activity (74–76). Wang et al. have found that a combination of mTOR inhibitor everolimus and metformin had a synergistic effect on inhibiting breast cancer cell growth (77). In a phase III randomized controlled study of early-stage breast cancer (78), metformin combined with a standard treatment showed significant overall survival and invasive DFS benefits over the placebo with a standard treatment in HER2-positive breast cancer (P = 0.038 and 0.0026, respectively). This indicates that TSC2 has significant potential in the treatment of HER2-positive breast cancer.

### 4.2 TSC2 in HR+/HER2- breast cancer

HR+/HER2- breast cancer accounts for approximately 60−75% of all breast cancers and is the most common breast cancer subtype. The ER pathway drives its development and progression and is directly and indirectly associated with TSC2 (79). Estrogen rapidly (within 5 min) stimulates TSC2 phosphorylation at T1462, one of the sites at which Akt phosphorylates and inactivates TSC2, suggesting that estrogen-induced cell proliferation is related to TSC2 inactivation (80). Some studies have also shown that TSC2 bound specifically to ERα and inhibited estrogen-induced proliferation by attenuating activation of platelet-derived growth factor receptor β and ERK1/2 (81). Therefore, it is clear that TSC2 plays an inhibitory role in the proliferation of cells in HR+ breast cancer.

In contrast to primary breast cancer (PBC), metastatic breast cancer (MBC) often has mutations in specific genes. Cha et al. have analyzed the data for 4,268 MBC and 5,217 PBC patients from eight different cohorts and found 11 genes containing *TSC2* that were more frequently changed in MBC samples from pan-metastatic sites (82). Savas et al. have also found that mutations in *TSC2* were significantly enriched in MBC compared to PBC (83). Lefebvre et al. have performed whole-exome sequencing of 216 tumor-blood pairs from MBC patients who provided a biopsy sample. There were 143 HR+/HER2- breast cancer samples, and *TSC2* was mutated in four (2.8%) of them. In contrast, according to the Cancer Genome Atlas data, the mutation rate of *TSC1*/*TSC2* was only 0.7% (P = 0.0004) in patients with HR+/HER2- early-stage breast cancer (84). This suggests that HR+/HER2- advanced breast cancer with a higher rate of *TSC2* mutations compared to HR+/HER2- early breast cancer may be associated with tumor progression or resistance to therapy.

PIK3CA mutations occur in nearly 40% of patients with HR+/HER2- breast cancer (85, 86). Inhibitors of PI3K p110α (PI3Kα) are highly effective in patients with PIK3CA-mutated tumors, especially in combination with endocrine therapy (85, 87). However, there is a subset of patients who remain resistant to PI3K inhibitors. It has been suggested that when PI3K is inhibited, 3-phosphoinositide-dependent protein kinase-1 can activate serum- and glucocorticoid-inducible kinase (SGK1), which is highly homologous to AKT. SGK1 overcomes PI3K inhibition *via* direct phosphorylation and inhibition of TSC2 to maintain mTORC1 activity (88). Cai et al. have analyzed 1,918 tumors from 1,756 breast cancer patients (89), of which 443 samples of ER+/HER2- breast cancer had PIK3CA mutations. They have found that about 1.35% of the ER+ tumors with PIK3CA mutations carried genomic alterations of *TSC2*, which may have led to resistance to PI3K inhibitors (90). In addition, Yuan et al. have analyzed 28,847 single nucleotide polymorphisms (SNPs) in 61 genes that participate in the mTOR pathway from 3,663 (1,983 ER+ and 1,098 ER-) breast cancer patients and 4,687 healthy women. The results have demonstrated that the *TSC2* gene was associated with the risk of overall and ER+ breast cancers (P = 0.009 and 0.012, respectively). However, *TSC2* SNP rs181088346 was significantly associated with a reduced overall risk of breast cancer (odds ratio = 0.77, 95% confidence interval = 0.65–0.88, P adj = 0.035) (91). Thus, the *TSC2* gene may be connected with genetic susceptibility of HR+/HER2- breast cancer.

### 4.3 TSC2 in TNBC

TNBC accounts for 10–20% of all breast cancers. It can be classified into the following subtypes based on multi-omics data: luminal androgen receptor (LAR) and its most noteworthy features associated with androgen receptor signaling; immunomodulatory (IM) with high expression of cytokine gene signaling and activation of immune cell signaling; basal-like and immune-suppressed with cell cycle upregulation, DNA repair activation, and immune response gene downregulation; and mesenchymal-like abundant in mammary stem cell pathways (92). The altered *TSC2* gene was observed in both metaplastic breast cancer (a rare special histological type of TNBC) and LAR cohorts (2/17, 2/8, respectively) (93, 94). TP53 is one of the most frequent mutated genes in TNBC somatic cells (95). Analyzing several data sets for breast cancer patients suggested that high expression of death-associated protein kinase 1 (DAPK1) is associated with a worse prognosis in patients with TP53-mutant cancers. Research has shown that DAPK1 overexpression increases the phosphorylation of TSC2 on Ser939, leading to the activation of the mTOR pathway (96). In addition, Huang et al. have found that TSC1/TSC2 inactivation may up-regulate programmed cell death receptor ligand 1 (PD-L1) at the transcriptional level and readjust the immune microenvironment by increasing the recruitment of cluster of differentiation (CD) 8^+^ T cells (97). In a case report, immunohistochemistry results for TNBC from a patient with *TSC2* mutation showed PD-L1 and CD8 T cells diffuse positivity (98). This suggested that loss-of-function TSC2 may alter the TNBC tumor microenvironment and that immunotherapy may be effective for patients with TNBC. In addition, the IM subtype expresses higher levels of immune-related genes, including PD1, PD-L1, and T lymphocyte-associated antigen 4. Immune-related markers with high levels of expression indicate that individuals with this type of tumor may benefit from using immune checkpoint inhibitors (92, 99). The benefits of immunotherapy have been demonstrated in several clinical studies, including Keynote-355 (100, 101).

## 5 mTOR inhibitors

TSC2 is the main negative regulatory protein of mTORC1. Mutation, down-regulation, and protein inactivation of *TSC2* in breast cancer cause over-activation of mTORC1, which leads to treatment resistance and metastasis. Therefore, it is extremely important to inhibit mTORC1. There are no drugs that directly target TSC2. However, an mTOR inhibitor (everolimus) has been approved by the U.S. Food and Drug Administration for treatment of breast cancer (102).

HR+/HER2- advanced breast cancer has a higher rate of TSC2 mutations compared to HR+/HER2- early breast cancer (83, 84). The BOLERO-2 study (103) has shown that everolimus combined with exemestane improved progression-free survival (PFS) compared to placebo and exemestane in postmenopausal patients with HR+ advanced breast cancer and resistance to aromatase inhibitor (AI). Everolimus also enhances the treatment efficacy of fulvestrant in AI-resistant, ER-positive metastatic breast cancer (104). In patients with AI-resistant HR+/HER2- breast cancer, the combination of everolimus with endocrine therapy has demonstrated a clinically significant benefit.

Miller et al. (105) have reported that HER2 inhibitor lapatinib reduces the cell growth and inhibits the activation of downstream effector mTORC1 in TSC2-expressing cells, but not in TSC2-knockdown cells. A combination of mTOR inhibition greatly improved the antitumor effects of trastuzumab (105). The BOLERO-3 study (106) has shown that the addition of everolimus to trastuzumab-based therapy in patients with HER2-positive breast cancer leads to a statistically significant prolonged PFS. Furthermore, everolimus in combination with antibody–drug conjugate T-DM1 had a synergistic antitumor effect in HER2-positive breast cancer (107). These outcomes suggested that the mTOR inhibitor plays a significant role in anti-HER2 therapy.

Everolimus is also used for patients with advanced TNBC. A Phase 1 trial for tamirolimus or everolimus in combination with doxorubicin and bevacizumab for patients with metastatic TNBC has shown that mTOR inhibitors extended the objective response rate to 21% and clinical benefit rate to 40%. Tissue samples were available for testing the genes in the PI3K pathway in 43 participants, but *TSC2* mutations were not detected (108). In addition, the mTOR inhibitor rapamycin was shown to decrease the PD-L1 expression in *PTEN*-mutant TNBC cell lines (109). Although conclusive clinical evidence is lacking, mTOR inhibition combined with immunotherapy may be effective in patients with TNBC (110).

## 6 Conclusion

TSC2 is the hub of multiple signaling pathway networks. It can regulate cell cycle, autophagy, and other physiological functions and is closely related to the development, treatment, and prognosis of breast cancer. In recent years, immunotherapy and targeted drug use, including CDK4/6 and PI3K pathway inhibitors, have been on the rise in breast cancer treatment. They are being continuously explored in combination with chemotherapy and endocrine therapy. TSC2 involves mechanisms of action of many antitumor drugs. In addition, low expression of TSC2 is associated with poor prognosis. Therefore, in-depth study of TSC2 functions provides important clinical guidance for improving treatment efficacy, overcoming drug resistance, and predicting prognosis. It can also suggest new directions for breast cancer drug development in the future.

## Author contributions

Q-YZ and Z-MH contributed equally to this work. Q-YZ and Z-MH wrote and edited the manuscript. W-MC and BL edited the manuscript and designed and supervised the work. All the authors read and approved the final version of the manuscript.
